# Sport-Specific Muscle Architectural Adaptations and Jump Performance in Preadolescent Rhythmic Gymnasts

**DOI:** 10.3390/children13030357

**Published:** 2026-02-28

**Authors:** Vasiliki Gaspari, Gregory C. Bogdanis, Ioli Panidi, Dimitra A. Kanna, Andreas Salagas, Anastasia Donti, Gerasimos Terzis, Olyvia Donti

**Affiliations:** School of Physical Education & Sport Science, National and Kapodistrian University of Athens, 17237 Athens, Greece; vgaspari@phed.uoa.gr (V.G.); gbogdanis@phed.uoa.gr (G.C.B.); ipanidi@phed.uoa.gr (I.P.); dimitrakanna@phed.uoa.gr (D.A.K.); andreassal@phed.uoa.gr (A.S.); adonti@phed.uoa.gr (A.D.); gterzis@phed.uoa.gr (G.T.)

**Keywords:** rhythmic gymnastics, fascicle length, muscle thickness, anatomical cross-sectional area, countermovement jump

## Abstract

**Objective:** We examined vastus lateralis (VL), gastrocnemius medialis (GM), gastrocnemius lateralis (GL), and biceps femoris (BF) muscle architecture and force–time parameters recorded during a countermovement jump (CMJ). **Methods:** Eighty-nine 9 year-old girls (43 rhythmic gymnasts and 46 recreationally active controls) were assessed in: (a) muscle architecture (fascicle length—FL; angle; muscle thickness; and anatomical cross-sectional area—CSA) using ultrasonography, (b) CMJ performance (maximum force—Fmax; rate of force development—RFD; jump height; and peak power) using force–time data, and (c) anthropometrics and body composition. **Results:** Rhythmic gymnasts exhibited greater BF fascicle length and muscle thickness than controls (7.84 ± 0.73 vs. 7.26 ± 0.75 cm and 1.76 ± 0.19 vs. 1.61 ± 0.22 cm, respectively, *p* < 0.001), while VL muscle CSA was larger in controls (*p* = 0.001). When normalized to the respective segment length (thigh or shank), the FL was longer in gymnasts across all muscles (*p* ≤ 0.017). Gymnasts also demonstrated greater CMJ height (13.1%, *p* = 0.005), power scaled to body mass, and RFD (*p* < 0.005), while controls produced a greater Fmax (16.9%, *p* = 0.002). Body mass was the strongest predictor of Fmax in both groups (*p* < 0.001). CMJ power was best predicted by gastrocnemius CSA in gymnasts and by VL CSA combined with maturity offset in controls (all *p* < 0.001). Maturity offset and gastrocnemius CSA also predicted allometrically scaled power in controls. **Conclusions**: Rhythmic gymnasts are characterized by muscle-specific adaptations, specifically in the BF muscle FL and muscle thickness, which favor superior CMJ performance. In developing athletes, body mass is primarily related to maximal force, whereas muscle CSA is more closely associated with power output.

## 1. Introduction

Skeletal muscle architecture—typically described by fascicle length (FL), pennation angle (PA), muscle thickness (MT), and anatomical cross-sectional area (CSA)—refers to the arrangement of muscle fibers within a muscle and is a key determinant of strength and power generation in adults [1]. Longer FLs enhance force production during high-velocity contractions [2], while increased MT and PA reflect more in-parallel sarcomeres and a greater physiological cross-sectional area, enhancing force capacity [3,4,5,6].

Muscle architecture develops substantially throughout childhood and adolescence due to growth, maturation, and mechanical loading [7,8,9,10]. Adolescents around 15 years of age tend to have longer FLs compared with younger children, reaching values comparable to adults [11,12]. PA appears to be muscle-specific; knee extensors show little change from childhood through adulthood [11,13], whereas the GM exhibits a developmental increase from birth until reaching a plateau after the adolescent growth spurt [14,15,16]. In addition, children have smaller MT than both adults [11,17] and older adolescents [13,17]. However, much of the existing evidence is derived from studies examining broad age ranges (e.g., 5–18 years) and mixed-sex samples, leaving gaps in understanding muscle architecture within specific developmental stages—particularly in preadolescent children and in females. Similarly, the extent to which architectural characteristics contribute to functional performance (e.g., sprinting, jumping) during childhood remains unclear. For example, Radnor et al. (2022) [10] reported that more biologically mature boys show greater MT, FL, and PA, alongside superior sprint and jump performance, indicating that muscle architecture contributes to performance. Notably, over 18 months, those reaching peak height velocity also demonstrated the greatest architectural adaptations and performance gains [10]. This suggests that periods of rapid growth may represent a heightened window for adaptation, where targeted lower limb training can build on natural maturational gains. However, current evidence is largely limited to boys, particularly adolescents [18,19]. In two recent studies comparing preadolescent and adolescent female volleyball athletes, it was found that child athletes exhibit shorter muscle fascicle lengths, smaller muscle cross-sectional areas, and lower muscle thicknesses compared with adolescent or post-PHV players [8,9]. In contrast, adolescents demonstrate greater gastrocnemius medialis and vastus lateralis architectural development, including longer fascicles and larger muscle sizes, particularly after peak height velocity [8,9]. In another cross-sectional study, Pentidis et al. [20] examined how maturation and gymnastics training affect muscle and tendon adaptation in prepubertal male artistic gymnasts. The findings indicated that, although systematic training in childhood improves strength and functional performance, clear hypertrophic changes in muscle or substantial increases in tendon stiffness were not observed at this developmental stage [20].

Despite the growing interest in muscle morphology during growth and maturation, evidence in preadolescent females remains limited—particularly when comparing trained young athletes with age-matched non-athletes. Regular athletic training loads the musculoskeletal system and initiates adaptation in muscles [21], tendons [22], and bones [23]. It is well established that muscle size, muscle strength, and tendon stiffness are greater in adolescent athletes compared to untrained controls of a similar age [24,25]. Due to substantial changes in muscle anabolic hormones [26] from early to late adolescence, muscle strength increases rapidly with a similar development in muscle size [27,28]. In childhood, though, the potential effects of exercise on muscle hypertrophy seem limited [29], and it is often suggested that training-induced muscle strength gains before puberty are more related to neural adaptations, which include changes in motor unit coordination, rate coding, and recruitment rather than muscle hypertrophy [30]. Given that some child athletes, such as rhythmic gymnasts, have already been training systematically (15–25 h of training per week) for several years, they may exhibit architectural adaptations that reflect this extensive loading from an early age and growth-related adaptations. Moreover, the relationships between muscle architectural characteristics and functional outcomes, such as force–time characteristics during lower limb power generation, has not been adequately examined. Therefore, the aim of this study was to compare muscle architecture adaptations of several lower limb muscles (VL, GM, GL, and BF) during long-term systematic training at a very early age in rhythmic gymnasts. In addition, we examined the association between muscle architecture and CMJ force–time parameters between child rhythmic gymnasts and controls. It was hypothesized that rhythmic gymnasts would exhibit greater fascicle length and muscle thickness in at least some muscles compared with controls and would demonstrate superior neuromuscular performance.

## 2. Materials and Methods

### 2.1. Subjects

Forty-three child rhythmic gymnasts (age: 9.16 ± 0.59 years) were recruited in collaboration with the National Gymnastics Federation. In addition, 46 age-matched recreationally active participants served as controls (age: 9.28 ± 0.61 years; sports: volleyball, basketball, tennis, track and field, and dancing). An a priori power analysis was conducted using G*Power (version 3.1). Based on previous studies with comparable experimental designs and sample sizes [31], a medium effect size (Cohen’s *d* = 0.60) was assumed. With an alpha level of 0.05 and a desired statistical power of 0.80 for a two-tailed independent samples *t*-test, the required total sample size was estimated at 45 participants (approximately 22–23 per group). At the time of data acquisition, rhythmic gymnasts trained six times per week for 4.5–5.00 h per session for the last year, while controls engaged in training 2–3 times per week for 1–1.5 h per session. The training of the rhythmic gymnasts included apparatus skills, plyometrics, landing, and balancing exercises. The characteristics of the participants are shown in Table 1.

All participants had no lower limb injuries in the past six months. Prior to participation, the athletes and their parents were fully informed about the study’s purpose and procedures and provided written informed consent. Additionally, participants were briefed on the potential risks and experimental procedures and gave their oral consent before taking part. All procedures were conducted in accordance with the principles of the Declaration of Helsinki and were approved by the local ethics committee (registration number: 1635/17-04-2024) in compliance with the World Medical Association Code of Ethics.

### 2.2. Experimental Design

**Figure 1:**
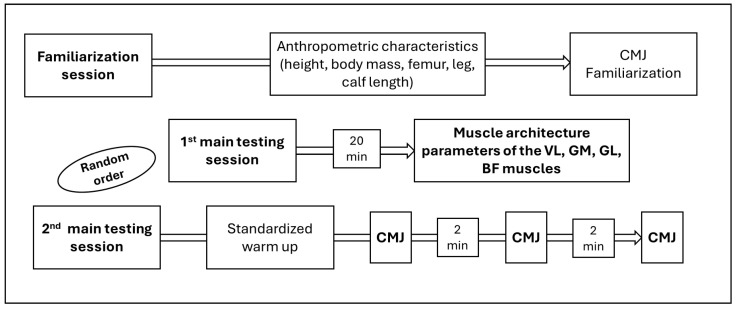
Schematic representation of the study protocol.

Participants completed one familiarization session followed by two main testing sessions, during which vastus lateralis (VL), gastrocnemius medialis (GM), gastrocnemius lateralis (GL), and biceps femoris (BF) muscle architecture, as well as CMJ force–time characteristics were assessed. During the familiarization session, participants’ anthropometric characteristics (height, body mass, femur, leg, and calf length) were measured, and they were familiarized with performing CMJs on the force platform. One week later, two main testing sessions were conducted 3–4 days apart in a randomized order (see Figure 1). In the first main session, muscle architecture at rest of the VL, GM, GL, and BF was measured. In the second session, participants performed three CMJs with 2 min rest intervals between jumps following a standardized warm-up. Participants were instructed to avoid strenuous activity for 24 h prior to testing, and all assessments were conducted in the morning.

### 2.3. Procedures

#### 2.3.1. Anthropometry and Body Composition

Height was measured using a stadiometer (Seca 208, GmbH & Co. KG, Hamburg, Germany), and body mass was recorded with a calibrated digital scale (Seca 710, GmbH & Co. KG, Hamburg, Germany). Body mass index (BMI) was calculated as body mass divided by the square of standing height (kg/m^2^). Femur length was measured as the distance between the greater trochanter and the lateral condyle of the femur. Leg length was determined as the distance from the greater trochanter to the floor while standing. Calf length was measured from the tibiofemoral joint to the most prominent point of the medial malleolus. Whole-body dual-energy X-ray absorptiometry (DEXA) was performed to assess body composition, quantifying bone mineral content and density, fat mass, and lean soft tissue mass (fat-free, non-bone tissue). In addition, bone mineral content and density, fat mass, and lean soft tissue mass were expressed as a percentage of total body mass to enable comparisons of their relative contributions to body composition between the two groups that differed in overall body mass (Table 1).

#### 2.3.2. Muscle Architecture Assessment

**Figure 2:**
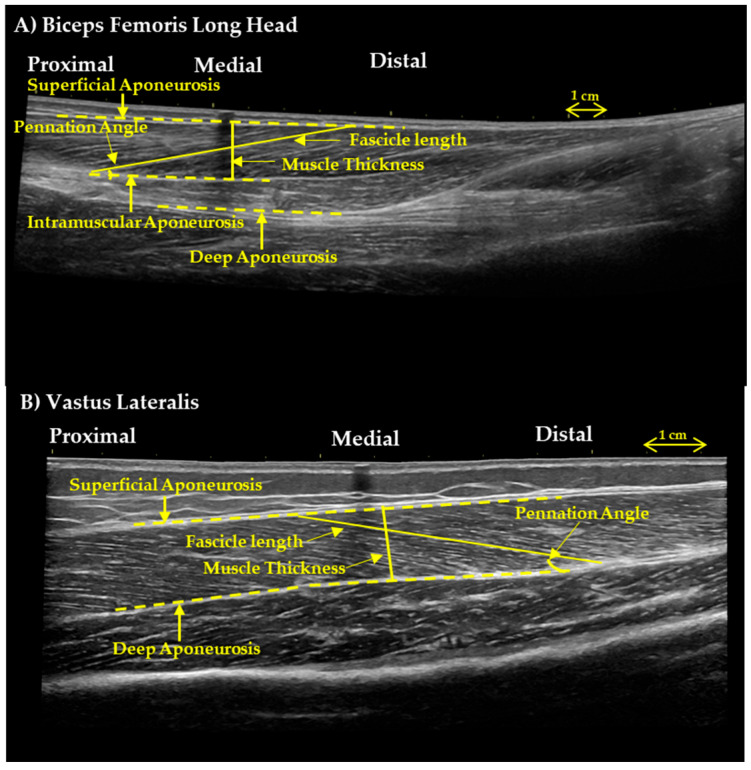
Panoramic ultrasound images of (**A**) biceps femoris long head and (**B**) vastus lateralis.

**Figure 3:**
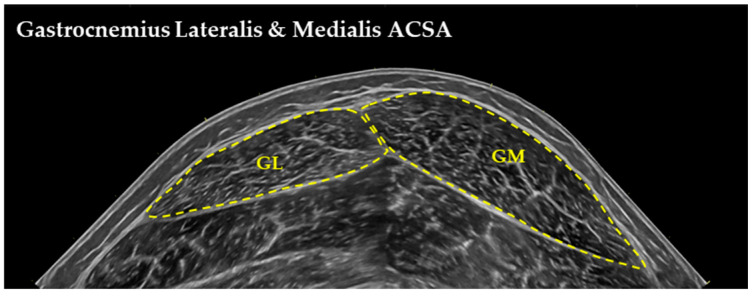
Anatomical cross-sectional area (CSA) of gastrocnemius lateralis (GL) and medialis (GM).

Ultrasound images were obtained in the morning, 24 h after the last training session. Participants rested in a supine position on a physiotherapy bed for 20 min with muscles relaxed, hips at 180°, and knees extended (~170°). VL architecture was measured at the midpoint of the muscle, 50% of the distance from the greater trochanter to the lateral femoral condyle [31]. GM and GL were measured at one-third (30%) of the distance from the popliteal crease to the center of the medial malleolus, and BF was measured at the midpoint (50%) from the ischial tuberosity to the fibular head. All measurements were performed on the right leg. Panoramic B-mode ultrasound images were acquired using a LOGIQ S9 system (General Electric, Boston, MA, USA) with an ML6-15 MHz linear array probe in extended field-of-view mode. Acoustic contact was maintained with Aquasonic clear gel (Parker Laboratories, New Fairfield, NJ, USA). The transducer was positioned longitudinally along the femur, parallel to the muscle fascicles, and perpendicular to the skin. A scanning path was marked on the skin with dashed lines (~10 cm) on either side of the 50% muscle length marker [32]. Images were analyzed using Motic Images Plus software (2.0, Motic, Hong Kong, China). For each participant, two FLs and their respective PAs were measured. MT was defined as the perpendicular distance between the superficial and deep aponeuroses and measured twice [33] (Figure 2). Anatomical cross-sectional area (CSA) was also assessed for VL, GM, GL, and BF, with average values used for analysis (Figure 3). FL of the lower limb muscles was scaled to respective bone length (e.g., tibia or femur length) to account for inter individual differences in skeletal dimensions and enable comparisons of muscle architecture across subjects (Table 1). Test–retest reliability was determined in 11 participants on two separate days. Intraclass correlation coefficient (ICC) for MT was 0.970 (90% CI: 0.913–0.990), for PA ICC was 0.942 (90% CI: 0.836–0.980), and for FL ICC was 0.948 (90% CI: 0.854–0.982).

#### 2.3.3. Countermovement Jump Performance

After a brief warm up consisting of 5 min on a stationary cycle ergometer at a standard load (60 W), followed by 5 min of dynamic stretching, participants performed three maximal countermovement jumps (CMJs) on a force platform with arms akimbo, resting for 2 min between jumps. The ascending portion of the force–time curve of the highest jump was used for further analysis. Variables extracted included CMJ Fmax, flight time, and minimum force (CMJ Fmin). In addition, variables were allometrically scaled to body mass (BM^0.67^) to account for the influence of body mass on force and power output [34]. Jumping height was calculated from take-off velocity, according to the following equation [35]: jumping height = (take-off velocity)^2^/(2 × *g*).

CMJ power and allometric power were also estimated according to the following equation [36]: Peak Power (W)= (61.9 × jump height) + (36 × body mass) + 1822.

CMJ Allometric power (W/kg^0.67^) = Peak power/body mass^0.67^.

Average rate of force development (ARFD) from minimum to maximum force and RFD at 0–50, 0–100, and 0–150 ms time intervals from minimum force (CMJ Fmin) were calculated according to the following equation: RFD (N × s^−1^) = ΔForce × ΔTime^−1^ [37].

This approach allowed for detailed assessment of both the magnitude and timing of force production during the CMJ.

### 2.4. Statistical Analysis

Means ± standard deviations (SDs) were calculated for all the examined variables. Data were tested for normality using the Kolmogorov–Smirnov test, with no notable violations observed. Independent-samples *t*-tests were used to examine differences between groups in muscle architecture and jumping performance. Cohen’s *d* was calculated to estimate effect sizes for pairwise comparisons. Pearson product–moment correlation (*r*) was used to assess relationships between muscle architecture and CMJ force–time parameters. Correlation strength was interpreted as: trivial (*r* < 0.10), small (*r* = 0.10–0.29), moderate (*r* = 0.30–0.49), large (*r* = 0.50–0.69), very large (*r* = 0.70–0.89), and almost perfect (*r* ≥ 0.90) [38]. Given the large number of correlation analyses performed in this study (n = 528), the likelihood of obtaining statistically significant results by chance alone is markedly increased, leading to an elevated risk of Type I error [39]. To control this inflation of the familywise error rate, the Bonferroni correction was applied [40], adjusting the significance threshold by dividing the α-level by the number of performed tests. After controlling for Type I error between correlations, stepwise regression analysis was used to examine the effect of muscle architecture and growth parameters on CMJ parameters according to the correlations that were maintained. Test–retest reliability was evaluated using intraclass correlation coefficients (ICCs, two-way mixed effects). Statistical significance was set at *p* < 0.05, and all analyses were conducted using SPSS (IBM SPSS Statistics, Version 25).

## 3. Results

### 3.1. Anthropometric Characteristics and Body Composition of the Participants

Rhythmic gymnasts were lighter, shorter, and were less biologically mature, as expressed by the maturity offset [41] compared with controls (Table 1). They also exhibited a significantly lower fat mass and lower total bone mass compared with the controls, while there were no group differences in lean body mass. However, when body composition was expressed relative to total body mass, gymnasts showed higher relative bone mass, lower relative fat mass, and greater relative lean mass compared with controls (Table 1).

### 3.2. Muscle Architecture

There were no significant between-group differences in FL, PA, and muscle thickness for the VL, GM, and GL muscles; however, the VL anatomical cross-sectional area was lower in gymnasts, while the BF fascicle length and thickness were higher in rhythmic gymnasts compared with controls (Table 2). Notably, rhythmic gymnasts exhibited greater relative FLs for VL, GM, GL, and BF normalized to the respective bone segment length than the controls (Table 2).

### 3.3. Countermovement Jump Force Time Parameters

Rhythmic gymnasts demonstrated higher CMJ height, greater power when scaled to body mass, longer flight time, higher take-off velocity, and faster rate of force development across the 0–100 ms and 0–150 ms intervals, whereas the controls produced greater maximal force (Table 3).

### 3.4. Correlations

In rhythmic gymnasts, body mass was positively correlated with CMJ power and maximal force but negatively correlated with allometrically scaled CMJ power. Gastrocnemius CSA was also positively associated with CMJ power and maximal force but was not significantly correlated with scaled power (Table 4). Only the correlations that remained statistically significant after applying the Bonferroni correction are presented in Table 4 and Table 5.

In controls, height, body mass, BMI, and maturity offset were positively correlated with absolute CMJ power and maximal force but negatively correlated with allometrically scaled CMJ power. Muscle architecture showed similar patterns, being positively associated with absolute CMJ power and maximal force and negatively associated with relative power measures (Table 5).

### 3.5. Stepwise Regression Analysis for Rhythmic Gymnasts

**Figure 4:**
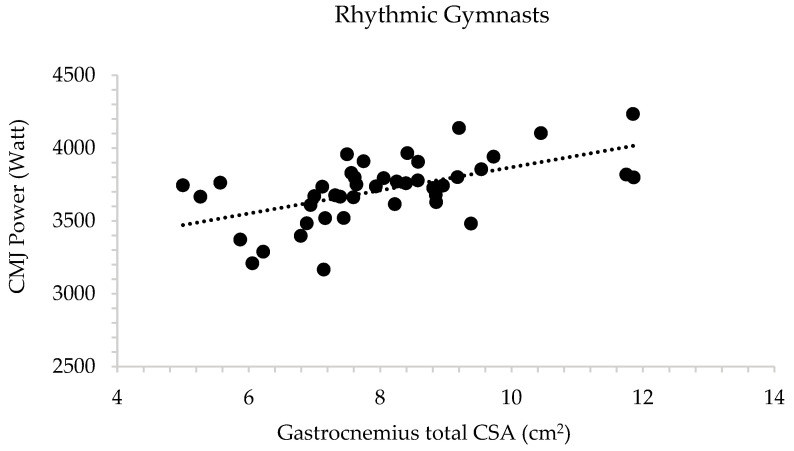
Multiple regression analysis examining the relationship between countermovement jump (CMJ) power and gastrocnemius cross-sectional area (CSA) in rhythmic gymnasts.

In rhythmic gymnastics athletes, stepwise regression analysis showed that gastrocnemius CSA accounted for a large part (30.2%) of the variance of CMJ power (adjusted R^2^ = 0.302, F = 19.129, and *p* < 0.001) (Figure 4). In addition, body mass accounted for a large part (39.1%) of the variance of CMJ Fmax (adjusted R^2^ = 0.391, F = 27.911, and *p* < 0.001).

### 3.6. Stepwise Regression Analysis for Controls

**Figure 5:**
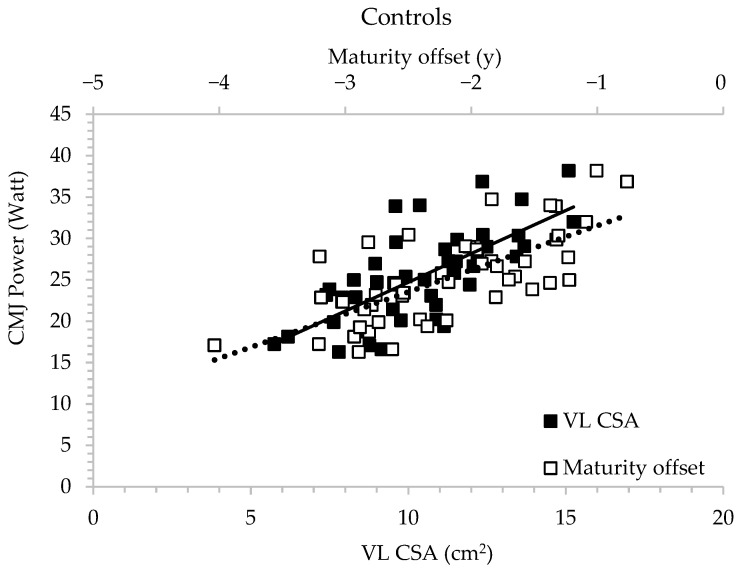
Multiple regression analysis examining the relationship between countermovement jump (CMJ) power, vastus lateralis cross-sectional area (VL CSA), and maturity offset in controls.

Maturity offset and VL anatomical cross-sectional area accounted for a large part (57.7%) of the variance of CMJ power (adjusted R^2^ = 0.557, F = 13.871, and *p* < 0.001) (Figure 5). In addition, maturity offset and gastrocnemius CSA accounted for (48.9%) of the variance of CMJ allometric power, although gastrocnemius CSA had a negative association (adjusted R^2^ = 0.489, F = 11.442, and *p* = 0.002). Body mass accounted for 47.4% of the variance of CMJ Fmax (adjusted R^2^ = 0.462 F = 39.572, and *p* < 0.001).

## 4. Discussion

The main finding of the study was that rhythmic gymnasts have greater biceps femoris FL and MT than age-matched controls. When normalized to the respective segment length, FL was longer in gymnasts across all muscles. Neuromuscular performance was also higher in gymnasts, as shown by the greater CMJ height, power scaled to body mass, and RFD. CSA of the gastrocnemius muscles was the main determinant of CMJ power in gymnasts, while in controls, the CSA of VL explained a considerable proportion of the variance in lower limb muscle power.

In the present study, rhythmic gymnasts displayed substantially greater biceps femoris FL and thickness, possibly reflecting the sport- and muscle-specific effects of gymnastics training on muscle architecture [42]. Measurable muscle architectural differences may emerge even in prepubertal children when they are exposed to intensive daily training (3–4 h per day) despite the generally limited hypertrophic response at this developmental stage. In addition, components of rhythmic gymnastics training, such as repeated hip extension and flexion movements and intensive stretching starting from a very young age, may represent the necessary load to attain longitudinal muscle architectural changes in the muscles involved. In the study of Panidi et al. [12], 12 weeks of intensive static stretching in adolescent female athletes resulted in significant increases in fascicle length at rest and during stretching. These findings suggest that repeated mechanical loading at long muscle lengths may stimulate mechanotransduction pathways, potentially contributing to muscle architectural remodeling and a hypertrophic response [12]. A previous cross-sectional study in 11-year-old preadolescent tennis players showed higher muscle hypertrophy in the dominant arm compared with age-matched non-athletes and attributed this to greater unilateral training volume [43]. Muscle architectural adaptations, such as a greater MT, are indicative of increased parallel sarcomere arrangement and may contribute to enhanced force production [20]. At the same time, longer fascicle lengths reflect a greater number of sarcomeres in series, which increases the shortening velocity and enhances performance in explosive stretch–shortening cycle movements [1,44].

When scaled to the respective segment length, FL was longer in rhythmic gymnasts compared with controls in all the examined muscles. Although there is limited evidence, some architectural characteristics seem to be related to systematic training in some sports. For example, the FL of the gastrocnemius medialis is longer in elite track-and-field sprinters and gymnasts compared to controls [32,42,45]. The selection of athletes with a favorable phenotype is important; however, the effects of long-term, systematic, and intensive sport-specific training may have a substantial impact on muscle architecture [8,9,22]. The few longitudinal static stretching studies have shown small increases in FL at rest and during stretching, coupled with reduced passive stiffness [45,46,47]. Our findings indicate that rhythmic gymnastics training may induce measurable muscle-specific adaptations, selectively affecting the BF and favoring longitudinal fascicle growth. In contrast, muscle PA and thickness in VL and gastrocnemius muscles were similar in gymnasts and controls, indicating that the training load of body weight-based skills in rhythmic gymnastics is insufficient to induce cross-sectional hypertrophic changes in VL [31,48].

As mentioned above, a functional consequence of longer FL is the faster shortening velocity and, thus, the power output [1,4]. Longer fascicles contain more sarcomeres in series, allowing for faster shortening and greater power production during explosive movements [49]. In addition, increased FL combined with greater MT in BF may also enhance force absorption and elastic energy storage during the eccentric phase of a jump, contributing to a more effective concentric propulsion. Collectively, these characteristics are related to greater CMJ performance and power, as observed in the present study. Together, longer fascicles (as scaled to segment length) and lighter body mass appear to support more effective neuromuscular performance, optimizing power production, jump height, and rapid force development—key requirements for the technical and explosive demands of rhythmic gymnastics. Accordingly, rhythmic gymnasts exhibited superior RFD, probably reflecting training-induced neural adaptations (including the speed and magnitude of motor unit recruitment, motor unit firing frequency, and intermuscular coordination [30,50]. In addition, superior jump performance in gymnasts may also be attributed to an enhanced jumping technique and intermuscular coordination developed through gymnastics training [50,51,52].

Body mass was the strongest predictor of CMJ Fmax in both groups, reflecting its contribution to maximal force production as well as its potential to represent sustained loading during growth [53]. Previous research has shown that body size and mass are key determinants of muscle strength in children and adolescents, with a larger body mass positively associated with a greater force-producing capacity [54]. Gastrocnemius CSA was the strongest predictor of CMJ power in gymnasts, whereas vastus lateralis CSA most strongly predicted CMJ power in controls, indicating potential differences in muscle contributions to jump mechanics between groups. Although quadriceps are major contributors to knee extension and force generation during the propulsive phase of vertical jumping, gastrocnemius contributes to plantar flexion and assists in the final push-off. Joint mechanics indicate that its activity and muscle–tendon behavior can influence propulsion and ankle mechanics, even though direct associations between its architecture and jump height are inconsistently reported [55]. Thus, the contribution of gastrocnemius size in gymnasts may reflect sport-specific distal push-off strategies, whereas a larger VL size in the controls aligns with quadriceps-dominant power generation. A large proportion of rhythmic gymnastics movements are performed in relevé (tiptoe) position, imposing substantial and repetitive loading on the ankle joint and plantar flexor muscles, which may promote functional adaptations and enhanced force-generating capacity at the ankle. Nonetheless, muscle CSA remained a strong predictor of power output in both groups, highlighting the role of muscle hypertrophy in force and power generation. These results are in line with previous findings in adolescent and adult populations, where thicker or more pennated muscles are associated with greater force and power output [4,56]. Moreover, Radnor et al. (2022) [10] also reported that FL and MT were significantly related to jump and sprint performance in boys undergoing maturation [10]. However, in the present study, these relationships were evident even in prepubescent children, indicating that morphological differences due to intensive training may precede or amplify maturational effects. Interestingly, in controls, CMJ power was predicted not only by vastus lateralis CSA but also by maturity offset, highlighting that both muscle size and biological maturation contribute to power development in the controls. In contrast, in gymnasts, gastrocnemius size alone predicted CMJ power, suggesting that chronic sport-specific training and muscle adaptations play a greater role than maturity, particularly in this group of younger and more homogenous athletes. It is possible that although maturation remains an important determinant of muscular performance in untrained children [57,58], targeted training can potentially override maturational influences in youth athletes exposed to specialized exercise stimuli at this age.

As expected, rhythmic gymnasts were lighter, shorter, and demonstrated delayed biological maturation compared with controls [57,59,60]. These characteristics may reflect both sport-specific selection criteria and inherent phenotypic traits that are advantageous for rhythmic gymnastics, in addition to training-related influences. Such attributes facilitate faster and more efficient rotational and flight movements during complex technical skills, which are fundamental to performance in this sport [29]. Rhythmic gymnasts also had lower body fat, which is consistent with the high energy demands and strict body composition demands of the sport [61]. In contrast, the relative lean body mass and bone mass were significantly higher in gymnasts than in controls (Table 1). Repeated exposure to strength and plyometric loading from a young age may induce musculoskeletal adaptations, including increased bone mineral density and a greater accumulation of lean body mass compared with non-athletic controls [57,59,61,62]. Thus, the initial selection process in rhythmic gymnastics may directly reflect differences in body dimensions and composition while also indirectly capturing differences in muscle architecture and potential adaptations; thus, hereditary predisposition should also be considered. The findings of this study have practical implications for rhythmic gymnastics coaches, highlighting how systematic training during childhood leads to muscle-specific adaptations selectively affecting BF and favoring longitudinal fascicle growth (when scaled to segment length), probably due to intensive flexibility and strength training at long muscle lengths. Importantly, although preadolescent strength gains are primarily attributed to neural factors, this research demonstrates that the muscle cross-sectional area—a proxy of hypertrophy—remains a key predictor of power output even in child female athletes. Therefore, coaches should implement developmentally appropriate strength and power training programs to optimize neuromuscular development and performance gains. Furthermore, this study demonstrates that intensive, high-volume training (15–25 h per week) can mitigate the effects of delayed biological maturation—commonly observed in gymnasts—enabling young gymnasts to attain superior neuromuscular performance compared to controls and favorable body composition. Increased lean mass and bone density result from early intensive training and support further athletic development.

## 5. Conclusions

Our findings suggest that rhythmic gymnastics training may elicit muscle-specific and sport-specific adaptations, selectively affecting the BF and favoring longitudinal fascicle growth (when scaled to segment length), accompanied by superior neuromuscular performance. The superior neuromuscular performance of rhythmic gymnasts is driven by both muscle architectural and neural factors due to sport-specific training adaptations. Furthermore, gymnastics training may favor bone and lean body mass adaptations despite the lower body mass and more delayed biological maturation. In all athletes, muscle power generation was primarily related to CSA, highlighting the role of muscle hypertrophy in power production.

### Limitations and Future Directions

A limitation of the present study is the cross-sectional design, which precludes causal conclusions about training-induced adaptations. Longitudinal studies examining changes in muscle architecture and performance throughout the pubertal transition are therefore warranted. Another consideration is that we did not measure hormonal and more direct growth-related factors, while maturation was assessed from anthropometric measurements and not from more direct methods such as skeletal age determination.

Another limitation of the study is the heterogeneity of the control group, as participants were involved in different types of general physical activities. However, they trained only 2–3 times per week for 1–1.5 h and did not engage in competitive or structured high-performance programs; thus, major sport-specific neuromuscular adaptations were unlikely. At the same time, this reflects typical recreational activity patterns in children, which enhances the external validity and general applicability of the findings to generally active pediatric populations.

## Figures and Tables

**Table 1:** Comparison between participants’ anthropometric characteristics (n = 89).

Anthropometrics and Body Composition	Rhythmic Gymnasts (n = 43)	Controls (n = 46)	Cohen’s *d*	*p*
Age (y)	9.16 ± 0.59 (9.01 to 9.32)	9.28 ± 0.61 (9.14 to 9.44)	0.20	0.341
Maturity offset (y)	−2.51 ± 0.53 (−2.64 to −2.38)	−2.17 ± 0.72 (−2.36 to −2.00)	0.54	0.002
Height (cm)	131.2 ± 5.8 (129.77 to 132.66)	135.8 ± 6.7 (134.07 to 137.48)	0.7	<0.001
Femur length (cm)	31.0 ± 2.0 (30.49 to 31.51)	32.7 ± 2.7 (32.02 to 33.37)	0.7	0.002
Calf length (cm)	29.3 ± 1.7 (28.86 to 29.74)	30.5 ± 2.3 (29.33 to 32.64)	0.6	0.005
Body mass (kg)	25.8 ± 3.0 (25.03 to 26.57)	31.1 ± 6.2 (29.56 to 32.64)	1.0	<0.001
BMI (kg/m^2^)	14.9 ± 1.1 (14.62 to 15.18)	16.7 ± 2.1 (16.18 to 17.22)	1.0	0.002
Fat mass total body (g)	5527 ± 1006 (5269 to 57,845)	9545 ± 3510 (8676 to 10,414)	1.5	<0.001
Lean mass total body (g)	19,313 ± 2466 (18,680 to 19,946)	20,409 ± 3065 (19,649 to 21,169)	0.4	0.071
Bone mass total body (g)	1023 ± 146 (985.5 to 1060.5)	1111 ± 192 (1064 to 1159)	0.5	0.018
Fat mass (% of body mass)	21.43 ± 3.15 (20.62 to 22.24)	29.95 ± 5.35 (28.63 to 31.29)	2.17	<0.001
Lean mass (% of body mass)	74.68 ± 3.13 (73.90 to 75.48)	66.25 ± 5.18 (64.97 to 67.53)	1.98	<0.001
Bone mass (% of body mass)	3.96 ± 0.28 (3.89 to 4.03)	3.60 ± 0.34 (3.52 to 3.68)	1.17	<0.001
Training characteristics				
Training hours per week (h)	23.77 ± 1.08 (23.49 to 24.05)	2.87 ± 0.34 (2.78 to 2.95)	14.9	<0.001
Training days per week (d)	6 ± 0 (6 to 6)	2 ± 0 (2.0 to 2.0)	16.0	<0.001
Training experience (y)	3 ± 1 (2.74 to 3.26)	2 ± 1 (1.75 to 2.25)	1.0	0.005

**Table 2:** Comparison between participants’ muscle architectural characteristics.

Muscle Architecture	Rhythmic Gymnasts (n = 43)	Controls (n = 46)	Cohen’s *d*	*p*
VL fascicle length (cm)	5.22 ± 0.64 (5.13 to 5.36)	5.24 ± 0.47 (5.07 to 5.38)	0.04	0.847
VL fascicle length/femur length	0.17 ± 0.01 (0.167 to 0.173)	0.16 ± 0.01 (0.158 to 0.162)	1.01	0.010
VL fascicle angle (°)	17.5 ± 2.0 (17.0 to 18.0)	17.7 ± 1.4 (17.4 to 18.1)	0.1	0.527
VL muscle thickness (cm)	1.57 ± 0.15 (1.53 to 1.61)	1.63 ± 0.22 (1.58 to 1.69)	0.32	0.198
VL CSA (cm^2^)	8.96 ± 1.54 (8.57 to 9.36)	10.35 ± 2.24 (9.80 to 10.90)	0.73	0.001
GM fascicle length (cm)	3.92 ± 0.44 (3.81 to 4.03)	3.82 ± 0.60 (3.67 to 3.97)	0.19	0.405
GM fascicle length/calf length	0.13 ± 0.01 (0.127 to 0.133)	0.12 ± 0.01 (0.118 to 0.122)	1.01	0.017
GM fascicle angle (°)	21.7 ± 2.8 (21.0 to 22.4)	21.0 ± 2.3 (20.4 to 21.6)	0.3	0.203
GM muscle thickness (cm)	1.49 ± 0.18 (1.44 to 1.54)	1.41 ± 0.22 (1.36 to 1.47)	0.40	0.086
GM CSA (cm^2^)	5.43 ± 1.18 (5.13 to 5.73)	5.20 ± 1.69 (4.78 to 5.62)	0.16	0.470
GL fascicle length (cm)	4.05 ± 0.45 (3.94 to 4.17)	3.92 ± 0.62 (3.77 to 4.07)	0.24	0.274
GL fascicle length/calf length	0.13 ± 0.01 (0.127 to 0.133)	0.12 ± 0.01 (0.118 to 0.122)	1.01	0.007
GL fascicle angle (°)	15.1 ± 1.9 (14.6 to 15.6)	15.5 ± 2.1 (15.0 to 16.0)	0.2	0.420
GL muscle thickness (cm)	1.07 ± 0.12 (1.04 to 1.10)	1.09 ± 0.17 (1.05 to 1.13)	0.14	0.566
GL CSA (cm^2^)	2.61 ± 0.60 (2.46 to 2.76)	2.73 ± 0.99 (2.49 to 2.98)	0.15	0.476
Gastrocnemius CSA (cm^2^)	8.03 ± 1.59 (7.62 to 8.44)	7.93 ± 2.57 (7.29 to 8.57)	0.05	0.825
BF fascicle length (cm)	7.84 ± 0.73 (7.65 to 8.03)	7.26 ± 0.75 (7.07 to 7.45)	0.79	<0.001
BF fascicle length/femur length	0.25 ± 0.02 (0.245 to 0.255)	0.22 ± 0.02 (0.215 to 0.225)	1.52	<0.001
BF fascicle angle (°)	12.6 ± 1.6 (12.2 to 13.0)	12.4 ± 1.9 (11.9 to 12.9)	0.1	0.575
BF muscle thickness (cm)	1.76 ± 0.19 (1.71 to 1.81)	1.61 ± 0.22 (1.56 to 1.67)	0.74	<0.001

**Table 3:** Comparison between participants’ countermovement jump force–time parameters.

CMJ Parameters	Rhythmic Gymnasts (n = 43)	Controls (n = 46)	Cohen’s *d*	*p*
CMJ height (cm)	15.51 ± 2.92 (14.76 to 16.26)	13.72 ± 2.93 (12.99 to 14.45)	0.62	0.005
CMJ Fmax (N)	667.76 ± 148.65 (629.62 to 705.90)	780.47 ± 181.21 (735.54 to 825.40)	0.69	0.002
CMJ allometric Fmax (N/kg^0.67^)	75.31 ± 13.23 (71.92 to 78.70)	78.09 ± 13.55 (74.73 to 81.45)	0.21	0.331
CMJ power (Watt)	3712.77 ± 221.65 (3655.89 to 3769.65)	3792.06 ± 274.21 (3724.16 to 3859.96)	0.32	0.143
CMJ allometric power (W/kg^0.67^)	422.14 ± 27.55 (415.07 to 429.21)	384.093 ± 32.98 (375.93 to 392.26)	1.26	<0.001
CMJ flight time (s)	0.36 ± 0.03 (0.35 to 0.37)	0.34 ± 0.03 (0.33 to 0.35)	0.67	0.006
CMJ take-off velocity (m/s)	1.77 ± 0.17 (1.73 to 1.81)	1.66 ± 0.18 (1.62 to 1.71)	0.63	0.006
CMJ 0–50 ms (N/s)	1282.14 ± 734.36 (1093.73 to 1470.55)	1743.62 ± 1402.90 (1396.22 to 2091.02)	0.41	0.058
CMJ 0–100 ms (N/s)	4085.52 ± 2859.61 (3351.66 to 4819.38)	5600.65 ± 3466.60 (4742.53 to 6458.77)	0.48	0.028
CMJ 0–150 ms (N/s)	7643.13 ± 3916.50 (6638.24 to 8648.02)	9519.11 ± 4423.47 (8423.96 to 10,614.26)	0.45	0.038
CMJ ARFD (N/s)	2984.88 ± 1985.30 (2475.45 to 3494.31)	3361.46 ± 2283.66 (2796.26 to 3926.66)	0.18	0.317
CMJ allometric ARFD ((N/s)/kg^0.67^)	334.38 ± 205.83 (281.56 to 389.7.20)	344.17 ± 244.42 (283.64 to 404.70)	0.04	0.839

**Table 4:** Correlations between anthropometric characteristics, muscle architecture, and jumping parameters in rhythmic gymnasts (n = 43).

	CMJ Power	CMJ Allometric Power	CMJ Fmax
Body mass	0.588 **	−0.647 **	0.636 **
Gastrocnemius CSA	0.564 **	−0.262	0.543

**: *p* ≤ 0.01.

**Table 5:** Correlations between anthropometric characteristics, muscle architecture, and jumping parameters in controls (n = 46).

	CMJ Power	CMJ Allometric Power	CMJ Fmax
Height	0.761 **	−0.595 **	0.455
Body mass	0.752 **	−0.836 **	0.688 **
Body mass index	0.567 *	−0.824 **	0.690 **
Maturity offset	0.648 **	−0.618 **	0.418
VL fascicle length	0.548 *	−0.500	0.445
VL muscle thickness	0.497	−0.560 *	0.406
VL CSA	0.664 **	−0.559 **	0.491
Gastrocnemius CSA	0.570 *	−0.569 *	0.581 **
BF fascicle length	0.571 *	−0.236	0.299
BF muscle thickness	0.625 **	−0.534	0.419
BF CSA	0.617 **	−0.611 **	0.639 **

*: *p* ≤ 0.05, **: *p* ≤ 0.01.

## Data Availability

Data are published in figshare: https://doi.org/10.6084/m9.figshare.31224010.
